# La varicelle n’est pas toujours bénigne

**DOI:** 10.11604/pamj.2018.31.30.16730

**Published:** 2018-09-13

**Authors:** Anass Ayyad, Maria Rkain, Abdeladim Babakhouya, Noufissa Benajiba

**Affiliations:** 1 Service de Pédiatrie, Centre Hospitalier Universitaire Mohamed VI, Faculté de Médecine et de Pharmacie, Université Mohammed Premier, Oujda, Maroc

**Keywords:** Varicelle-zona, maladie éruptive, complication, pleuropneumopathie, Varicella zoster, rash illness, complication, pneumonia with empyema

## Abstract

La varicelle est une maladie éruptive due au virus varicelle-zona qui touche essentiellement les enfants et dont l'évolution est habituellement bénigne. Cependant des complications de gravité variable peuvent être observées tel que les complications infectieuses bactériennes et les complications neurologiques. Nous rapportons deux observations de varicelle compliquée. Comme première observation, un nourrisson de 5 mois sans antécédents qui présente depuis six jours une éruption cutanée fait de vésicules et pustules, la symptomatologie s'est aggravée la veille de son admission au service par l'installation d'une détresse respiratoire. Comme deuxieme observation, une fillette de 7 ans, admise au service pour prise en charge d'une convulsion simple, et chez qui l'examen clinique a objectivé des cicatrices de varicelle généralisées et une ataxie cérébelleuse. Bien que la varicelle soit connue comme une affection virale commune, le plus souvent bénigne, plusieurs études ont récemment fait état d'une recrudescence de ses complications, qui semblent responsables de 0,2 à 1,5% des motifs d'hospitalisation des enfants atteints de varicelle.

## Abstract (en)

Varicella is a rash illness caused by the varicella-zoster virus. It mainly affects children with usually a benign outcome. However, several complications of variable severity can be observed including bacterial infectious complications and neurological complications. We report two cases of complicated varicella. Case 1: 5 month old baby with no previous pathological history presenting with a rash composed of vesicles and pustules lasting for six days. Symptoms worsened the day before his admission to our Department due to respiratory distress. Case 2: 7-Year old girl admitted to our Department due to simple convulsion. Clinical examination showed generalized varicella scars and cerebellar ataxia. Although varicella is a common, in most cases benign viral disease, several studies have recently reported a recrudescence of complications, which appear to be responsible for 0.2-1.5% of the causes of hospitalization in children with varicella.

## Introduction

La varicelle est une maladie éruptive causée par le virus varicelle-zona qui touche essentiellement les enfants vers l'âge préscolaire et dont l'évolution est généralement bénigne. Néanmoins de nombreuses complications sont rapportées, affectant environ 3 à 5% des malades et responsables d'une mortalité estimé à 1,4/100 000 et qui, paradoxalement, affecte surtout les sujets immunocompétents [1-3]. Les complications infectieuses bactériennes restent les plus fréquentes et sont dues principalement au staphylocoque et au streptocoque. Les complications neurologiques types occupent le second rang et sont dominées par la cérebellite habituellement bénigne tandis que la méningo-encéphalite est beaucoup plus rare mais plus gave. Les pleuropneumopathies sont rares chez l'enfant, sauf le nourrisson < 6 mois mais représentent une cause non négligeable de mortalité [1]. De nombreuses complications sont par ailleurs rapportées avec une faible fréquence telle que les atteintes hépatique, hématologique, cardiaque et articulaire.

## Patient et observation

**Figure 1:**
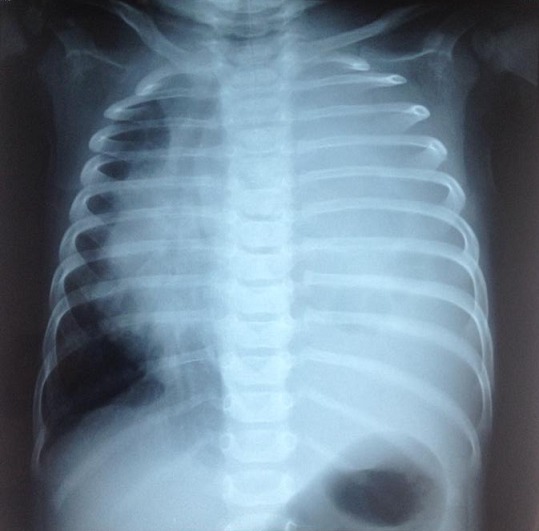
Radiographie thoracique de face montrant une pleurésie à gauche

**Figure 2:**
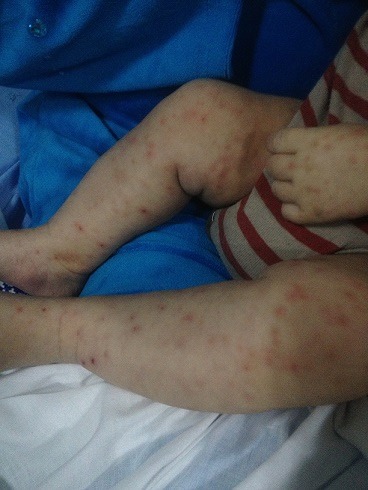
Lésions de varicelle d'âge différent

Nourrisson de 5 mois sans antécédents pathologique notable présentant une varicelle depuis 6 jours associée à une fièvre chiffrée entre 38, 5-39°C, la veille de son admission la symptomatologie s'est aggravée par l'installation d'une détresse respiratoire, des accès de cyanose péribuccale avec une fièvre chiffrée à 40°C et résistante au traitement antipyrétique puis le patient a présenté un épisode de convulsion raison pour laquelle il a été amené en consultation. L'examen clinique à l'admission a trouvé: un nourrisson geignard, légèrement hypotonique, fébrile à 39,5°C, polypnéique 50 cycles/min avec une SaO^2^; à 88% en aire ambiant, et à l'examen dermatologique des vésicules et pustules d'âge différent sur fond érythémateux généralisées (Figure 1). Le bilan biologique a montré un syndrome infectieux: CRP à 59mg/L, une hyperleucocytose à polynucléaire neutrophile (globules blancs à 26 850; 60% de PNN et 21% de lymphocytes). La radiographie du thorax (Figure 2) et l'échographie ont montrés une pleurésie de grande abondance gauche avec refoulement du médiastin et du poumon droit. L'étude du liquide céphalorachidien a montré une leucocytose à 60 éléments/mm³ avec une prédominance lymphocytaire à 80%, et la culture du liquide pleurale était négative. Le nourrisson a bénéficié un drainage pleural, une antibiothérapie à large spectre probabiliste, un traitement antiviral à base d'acyclovir et une oxygénothérapie. L'évolution était bonne, on a noté une amélioration des signes cliniques, biologiques et radiologiques. La durée d'hospitalisation était de 25 jours. Fillette de 7 ans, qui présente comme antécédents une varicelle 7 jours auparavant. Admis au service pour prise en charge d'une convulsion simple, et chez qui l'examen clinique a objectivé une fièvre à 38,1°C, des cicatrices de varicelle généralisées et une ataxie cérébelleuse. L'imagerie par résonance magnétique cérébrale était sans anomalie ainsi que l'étude du liquide céphalorachidien, par ailleurs la CRP était à 20mg/l; le diagnostic d'une cérebellite varicelleuse a été retenu; l'évolution a été bonne et le suivi a objectivé chez elle l'apparition d'une boulimie avec une importante prise pondérale.

## Discussion

Plusieurs études concernant l'évaluation des complications de la varicelle ont été élaborée et un certain nombre de facteurs de risque de varicelle grave ou compliquée ont été identifiés: l'âge représente le facteur essentiel. L'enfant de moins de 5 ans présente un risque élevé de complications, notamment infectieuses, et la mortalité chez le nourrisson de moins d'un an est 4 fois plus é1evée que chez les enfants plus âgés [1, 3, 4-6]. Les surinfections bactériennes cutanées et des tissus mous restent les plus fréquentes des complications dont la plus grave étant la fasciite nécrosante, Les germes en cause sont le plus souvent: le staphylococcus aureus et le streptococcus pyogenes [3]. Les complications neurologiques viennent au second rang et sont dominées par les cérebellite dont l'évolution est bénigne vers la résolution spontanée en quelques jours à quelques semaines tel est le cas pour l'observation 2, les méningo-encéphalites sont rare leurs incidence est estimé à de 1,7 à 4/10 000 aux États-Unis [2] et présente une tout autre gravité puisqu'elle est une cause significative de mortalité, notamment chez les jeunes nourrissons, le syndrome de reye et de plus en plus moins fréquent. Contrairement à l'adulte Les pneumopathies représentent une complication rare chez l'enfant sans facteur de risque particulier, sauf chez le nourrisson de moins de 6 mois chez qui elles représentent la cause prédominante de mortalité [3, 7]. En fait, il convient de distinguer les surinfections bactériennes, sous forme de pneumopathie ou de pleuropneumopathie, le plus souvent à pneumocoque, streptocoque hémolytique ou staphylocoque, largement majoritaires, surtout chez les sujets antérieurement sains et les pneumopathies interstitielles directement liées au virus de la varicelle et survenant le plus souvent chez des immunodéprimés [1, 2, 7].

## Conclusion

Les complications de la varicelle sont parfois grave voir même mortelles surtout chez les enfants âgés de 5 ans et moins et plus précisément le nourrisson de J1 à une année de vie, de ce fait il faut jamais hésiter à hospitalisé tout enfant ayant un facteur de risque et/ou des signes cliniques neurologiques, pulmonaires ou de surinfection bactérienne grave.

## Conflits d’intérêts

Les auteurs ne déclarent aucun conflit d'intérêts.
